# The Effect of Intraoperative Fentanyl Consumption on Prognosis of Colorectal Liver Metastasis treated by Simultaneous Resection: A Propensity Score Matching Analysis

**DOI:** 10.7150/jca.74674

**Published:** 2022-08-29

**Authors:** Yizhou Zhang, Qichen Chen, Xiao Chen, Mingzhu Zhang, Peng Li, Zhen Huang, Hong Zhao, Hongliang Wu

**Affiliations:** 1 Department of Hepatobiliary Surgery, National Cancer Center/National Clinical Research Center for Cancer/Cancer Hospital, Chinese Academy of Medical Sciences and Peking Union Medical College, 100021, Beijing, China.; 2 Department of Anesthesiology, National Cancer Center/National Clinical Research Center for Cancer/Cancer Hospital, Chinese Academy of Medical Sciences and Peking Union Medical College, 100021, Beijing, China.

**Keywords:** Colorectal liver metastasis, fentanyl, opioids, prognosis, recurrence

## Abstract

**Background:** No previous studies have reported the effect of intraoperative opioid consumption in colorectal liver metastasis (CRLM).

**Methods:** Medical records of patients who received simultaneous resection of CRLM were retrospectively reviewed. Patients with epidural anesthesia, intraoperative morphine, or intraoperative oxycodone were excluded. Patients were separated into high- and low-dose groups by median intraoperative equianalgesic fentanyl dose. Short-term outcomes, progression-free surcical (PFS) and overall survival (OS) were compared between groups before and after 1:1 propensity score matching (PSM). Univariable and multivariable Cox regression analysis were performed to identify independent predictors of survival.

**Results:** The final study population included 343 patients. Patients were separated into the low dose group (n=172) and the high dose group (n=171) by median intraoperative equianalgesic fentanyl dose (8.33 μg/kg). After PSM, 55 patients in the low dose group were matched to 55 patients in the high dose group and the baseline characteristics of the two groups were balanced. The two groups had no statistically significance difference in severity and categories of postoperative complications before and after PSM. Before PSM, the two groups had similar PFS (median 10.2 vs. 12.4 months, *P*=0.54) and OS (median 59.0 vs. 58.3 months, *P*=0.76). Univariate and multivariate Cox regression analyses revealed no statistically significant association between intraoperative equianalgesic fentanyl and PFS (multivariate HR=0.852, 95% CI 0.655-1.11, *P*=0.235) and OS (multivariate HR=1, 95% CI 0.68-1.49, *P* = 0.981). After PSM, the two groups also had similar PFS (median 9.2 vs. 10.7 months, *P*=0.98) and OS (median 51.0 vs. 46.0 months, *P*=0.39). Univariate and multivariate Cox regression analyses revealed no statistically significant association between intraoperative equianalgesic fentanyl and PFS (multivariate HR=1.05, 95% CI 0.632-1.73, *P*=0.861) and OS (multivariate HR=1.74, 95% CI 0.892-3.38, *P* = 0.105).

**Conclusion:** Intraoperative opioids consumption was not correlated with outcomes of CRLM patients treated with simultaneous resection.

## Introduction

Opioids are the mainstay of analgesics in surgery and postoperative pain control of cancer. In recent years, clinicians were concerned that the use of opioids might promote tumorigenesis, cancer metastasis, or recurrence 1. One possible mechanism is that opioids were long known to inhibit the activity of natural killer (NK) cells, the crucial component of antitumor immunity, in a dose-dependent manner 2, 3. A large number of clinical studies have focused on the effect of perioperative opioid consumption on the prognosis of patients, and yielded conflicting results for various types of cancer 4-9. Despite the great heterogeneity in the design and settings of these studies, the inconsistent results of these studies suggest that the prognostic effect of opioid consumption may depend on the type of malignancy. The expression of opioid receptors in certain types of cancer cells might present an alternative pathway for opioids to affect cancer progression and prognosis 10. Thus, specific clinical studies were required to investigate the role of opioids in a particular type of cancer.

Previous clinical studies on colorectal cancer also yielded inconclusive results. Increased opioids consumption were correlated with worse prognosis in a study of metastatic or recurrent colorectal cancer 11, while other studies on colorectal cancer treated with surgeries found no correlation between perioperative opioids consumption on patient prognosis 4, 12. These results indicated that the effect of opioids consumption on patients with colorectal cancer might depend on stage of the disease. In addition, preclinical study suggested that opioids could promote metastatic abilities of human colorectal cancer cells 13. Therefore, colorectal cancer patients in metastatic stage disease may respond differently to opioids. More than 50% of colorectal cancer patients would develop colorectal liver metastasis (CRLM) in their lifetime, and approximately 20% of patients developed liver metastasis at diagnosis, termed synchronous CRLM 14. Surgical resection is currently the treatment of choice for CRLM. With complete surgical resection, about one-sixth of CRLM patients can be cured 15. The effect of perioperative opioids consumption on prognosis of patients with CRLM is still poorly understood, as previous studies on colorectal cancer excluded stage IV patients 4, enrolled inadequate number of patients with CRLM 12, or focused on unresectable patients 11. No previous studies specifically investigated the effect of perioperative fentanyl consumption on prognosis of CRLM patients.

The aim of this study was to investigate the effect of intraoperative fentanyl consumption on the prognosis of CRLM patients treated with simultaneous resection.

## Methods

### Study population

This study focused on CRLM patients treated with simultaneous resection for: (1) simultaneous surgical resection of primary tumor and liver metastasis is the preferred surgical approach for synchronous CRLM at this institution; (2) patients treated with staged surgeries might experience opioid tolerance or opioid-induced hyperalgesia, which make it difficult to investigate the effect of intraoperative opioids on prognosis. We retrospectively reviewed medical records of patients who received simultaneous resection of CRLM at Cancer Hospital, Chinese Academy of Medical Sciences between December 2008 and May 2019. Patients receiving simultaneous surgery at this institution are required to be Child-Pugh A, while emergency surgery for symptomatic primary tumour is considered as a contraindication of simultaneous resection. Rectal resection/low rectal resection is not considered as a contraindication of simultaneous resection. Major hepatic resection (resection of more than 2 liver segments) is also not considered as a contraindication of simultaneous resection. Only adult patients who received surgery for a curative intent were included in this study. Patients with incomplete medical records were excluded from this study. We further excluded patients with epidural anesthesia, as a reliable approach to calculate the equianalgesic dose of epidural opioids was lacking. Finally, we excluded patients with intraoperative morphine or oxycodone from analysis, as dose conversion between opioids had been controversial 16, and these two opioids were only given to a small proportion of patients who received simultaneous resection of CRLM intraoperatively at this institution. The study was conducted in accordance with the Declaration of Helsinki (as revised in 2013). Ethics approval of this study was obtained from the Institutional Review Board of the Cancer Hospital, Chinese Academy of Medical Sciences (ID:NCC2019C-016) and individual consent for this retrospective analysis was waived.

### Anesthetic and analgesic methods

Patients who received simultaneous resection of CRLM generally underwent combined general anesthesia at this institution. The doses of strong opioids (fentanyl, sufentanil, remifentanil, morphine, oxycodone) were retrieved from surgical records of each patient, and the total dose during the operation was calculated. The doses of sufentanil and remifentanil were converted to equianalgesic fentanyl dose by the following manner: 0.1 μg sufentanil for 1 μg equianalgesic fentanyl, 1 μg remifentanil for 1 μg equianalgesic fentanyl. Intraoperative equianalgesic fentanyl dose per kilogram body weight is the exposure variable of this study.

### Follow-up and Outcomes

Postoperative complications were recorded until hospital discharge. The Clavian-Dindo classification system is applied to classify postoperative complications by severity 17. Major complication is defined as Clavian-Dindo grade III or IV. For patients with multiple postoperative complications, complication with the highest grade was recorded. Postoperative complications were further classified into general complications and surgery-related complications. Anastomotic leak, gastrointestinal tract necrosis, intrathoracic or intraabdominal abscess, hemorrhage, and ileus were considered surgery-related complications while other complications were considered as general complications 18. Hypertension and diabetes were recorded as comorbidities. As described previously, patients were followed up at regular intervals 19. In brief, the initial follow-up was performed 1 month after surgery, then follow-ups were performed every 3 months thereafter. Progression-free survival (PFS) was defined from surgery to detection of tumor progression or the last follow-up. Overall survival (OS) was defined from surgery to death or the last follow-up.

### Statistical analyses

Categorical variables were presented as percentages and compared by the Chi-square test. Continuous variables were present as median and interquartile ranges (IQR) and compared by the Mann-Whitney U test. The median intraoperative equianalgesic fentanyl dose was selected as the cut-off to separate patients into high- and low-dose groups accordingly. Survival was analyzed by the Kaplan-Meier method and compared by the log-rank test. Potential predictors of survival identified in the univariate analyses with *P*<0.10 and variable of interest (intraoperative equianalgesic fentanyl consumption) entered subsequent multivariate Cox regression analysis. To adjust for differences in baseline characteristics between groups, we performed 1:1 propensity score matching (PSM) by the 'nearest' method and without replacement. PSM was performed with the 'MatchIt' package of the R software. Two-sided *P*<0.05 indicates statistical significance. All statistical analyses were performed by the R software (Version 4.0.2).

## Results

### Study population

**Figure 1:**
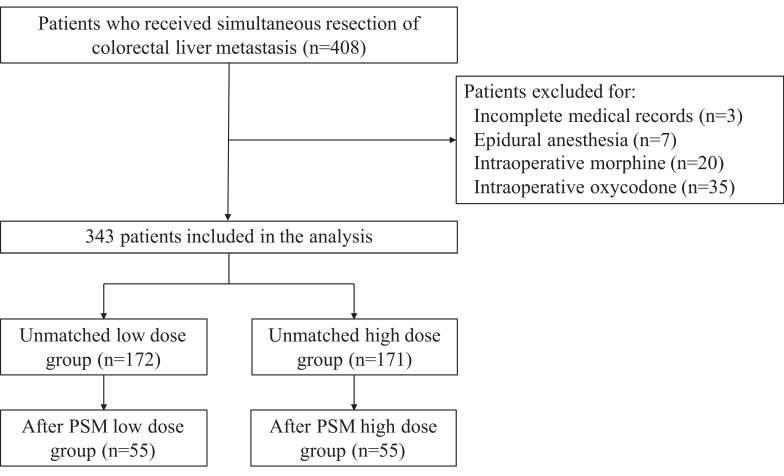
Flow diagram.

Medical records of 408 patients who received simultaneous resection of CRLM were retrospectively reviewed. Three patients were excluded for incomplete medical records, 7 patients were excluded for epidural anesthesia, and 55 patients were excluded for intraoperative morphine or oxycodone. Three hundred and forty-four patients remained in the final study population. The flow diagram of this study was shown in **Figure 1**. Each patient in the study population received at least one of fentanyl, sufentanil, or remifentanil intraoperatively. The median intraoperative equianalgesic fentanyl dose per kilogram body weight was 8.33 (6.53-14.0) μg/kg. The low-dose group consists of 172 patients with equianalgesic fentanyl dose≤8.33 μg/kg, while the high-dose group consists of 171 patients with equianalgesic fentanyl dose>8.33 μg/kg. Patient demographics, clinicopathological characteristics, and surgery and chemotherapy details were listed in **Table 1**. The low dose group had higher BMI (median 24.10 vs. 23.31, *P*=0.004), higher proportion of patients with comorbidities (51.7% vs. 36.8%, *P*=0.005), higher proportion of patients with ASA III (17.4% vs. 7.0%, *P*=0.013), and higher CEA (median 10.14 vs. 6.75 ng/μL, *P*=0.005).

To adjust for differences in baseline characteristics between groups, we performed 1:1 PSM. After PSM, 55 patients in the low dose group were matched to 55 patients in the high dose group. The baseline characteristics were well-balanced between the two groups (**Table 1**).

### Short-term outcomes

Details of short-term outcomes were listed in **Table 2**. Before PSM, the two groups were comparable in length of postoperative hospital stay (median 10 vs. 10 days, *P*=0.841). When classified into major complications and minor complications according to the Clavian-Dindo scoring system, a trend of the low dose group having lower proportion of no (48.3% vs. 54.4%) or minor (23.8% vs. 27.5%) complications and higher proportion of major complications (27.9% vs. 18.1%) was observed (*P*=0.099). The categories of postoperative complications were comparable (*P*=0.615). After PSM, the low dose group had numerically longer postoperative hospital stay (median 11 vs. 9 days, *P*=0.199). A trend of the low dose group having lower proportion of no (43.6% vs. 52.7%) or minor (23.6% vs. 32.7%) complications and higher proportion of major complications (32.7% vs. 14.5%) was observed (*P*=0.077). The categories of postoperative complications were comparable (*P*=0.272).

### Progression-free survival and overall survival

**Figure 2:**
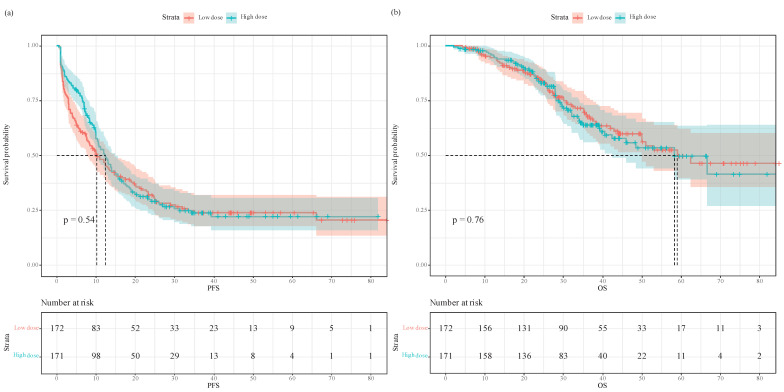
Progression-free survival and overall survival in patients who received low- versus high-dose intraoperative equianalgesic fentanyl before propensity score matching: **(a)** Progression free survival; **(b)** overall survival.

In the unmatched full cohort, the Kaplan-Meier survival plot and log-rank test revealed the two groups having comparable PFS (**Figure 2a**, median 10.2 vs. 12.4 months, *P*=0.54) and OS (**Figure 2b**, median 59.0 vs. 58.3 months, *P*=0.76). In univariate analysis, intraoperative equianalgesic fentanyl (high dose vs. low dose) was not associated with PFS (HR=0.925, 95% CI 0.720-1.19, *P*=0.541). In subsequent multivariate analysis, intraoperative equianalgesic fentanyl (high dose vs. low dose) was not associated with PFS (HR=0.852, 95% CI 0.655-1.11, *P*=0.235). Primary lymph node metastasis (HR=1.84, 95% CI 1.32-2.56, *P*<0.001), extrahepatic metastasis (HR=1.97, 95% CI 1.3-2.99, *P*=0.001), R0 resection (HR=0.686, 95% CI 0.509-0.924, *P*=0.013), and major hepatic resection (HR=1.49, 95% CI 1.04-2.12, *P*=0.029) were independent predictors of PFS.

In univariate analysis, intraoperative equianalgesic fentanyl (high dose vs. low dose) was not associated with OS (HR=1.06, 95% CI 0.732-1.53, *P*=0.765). In subsequent multivariate analysis, intraoperative equianalgesic fentanyl (high dose vs. low dose) was not associated with OS (HR=1, 95% CI 0.68-1.49, *P*=0.981). Age (HR=1.04, 95% CI 1.02-1.06, *P*=0.002), number of liver metastasis (HR=1.13, 95% CI 1.02-1.24, *P*=0.015), primary tumor T3 or T4 (HR=3.44, 95% CI 1.06-11.1, *P*=0.040), primary lymph node metastasis (HR=3.04, 95% CI 1.65-5.58, *P*<0.001), adjuvant chemotherapy (HR=0.484, 95% CI 0.324-0.725, *P*<0.001), and major hepatic resection (HR=1.84, 95% CI 1.06-3.19, *P*=0.032) were independent predictors of OS.

**Figure 3:**
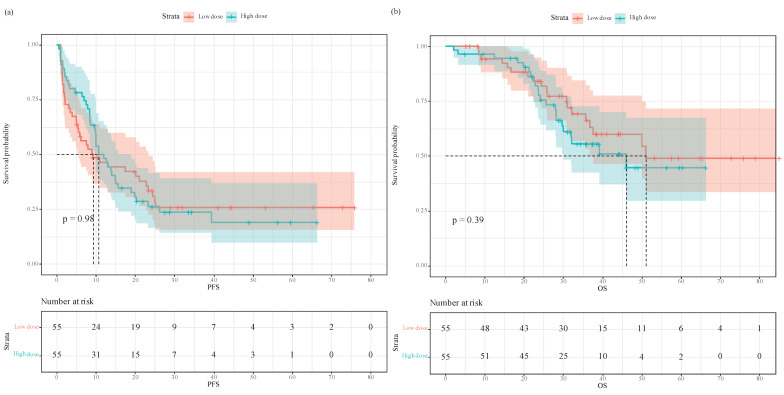
Progression-free survival and overall survival in patients who received low- versus high-dose intraoperative equianalgesic fentanyl after propensity score matching: **(a)** Progression free survival; **(b)** overall survival.

After propensity score matching, the Kaplan-Meier survival plot and log-rank test revealed the two groups having comparable PFS (**Figure 3a**, median 9.2 vs. 10.7 months, *P*=0.98) and OS (**Figure 3b**, median 51.0 vs. 46.0 months, *P*=0.39). In univariate analysis, intraoperative equianalgesic fentanyl (high dose vs. low dose) was not associated with PFS (HR=0.996, 95% CI 0.639-1.55, *P*=0.984). In subsequent multivariate analysis, intraoperative equianalgesic fentanyl (high dose vs. low dose) was not associated with PFS (HR=1.05, 95% CI 0.632-1.73, *P*=0.861). Primary lymph node metastasis (HR=2.42, 95% CI 1.34-4.36, *P*=0.004), extrahepatic metastasis (HR=2.23, 95% CI 1.07-4.67, *P*=0.033), and neoadjuvant chemotherapy (HR=0.588, 95% CI 0.364-0.951, *P*=0.031) were independent predictors of PFS.

In univariate analysis, intraoperative equianalgesic fentanyl (high dose vs. low dose) was not associated with OS (HR=1.31, 95% CI 0.706-2.43, *P*=0.392). In subsequent multivariate analysis, intraoperative equianalgesic fentanyl (high dose vs. low dose) was not associated with OS (HR=1.74, 95% CI 0.892-3.38, *P*=0.105). Age (HR=1.06, 95% CI 1.02-1.11, *P*=0.003), primary lymph node metastasis (HR=2.83, 95% CI 1.14-7.02, *P*=0.025), and intraoperative blood loss (mL) (HR=1.001, 95% CI 1-1.003, *P*=0.035) were independent predictors of OS.

## Discussion

This is the first study to evaluate the effect of intraoperative opioid consumption on outcomes of CRLM patients. In this study, we demonstrated that for patients who received simultaneous resection of CRLM, intraoperative equianalgesic fentanyl dose was not associated with postoperative complications, PFS, or OS before and after PSM.

Previous clinical studies evaluating the prognostic effect of perioperative opioid consumption have associated increased opioid dose with a higher risk of developing disease recurrence in patients with non-small-cell lung cancer (NSCLC) 6, esophagus squamous cell carcinoma 20, and laryngeal squamous cell carcinoma 21. Several plausible mechanisms were raised to explain the correlation between increased perioperative opioid consumption and a higher risk of disease recurrence in these types of cancer. First, opioids were known to have immunosuppressive effects, especially by inhibiting the activity of NK cells 3, 22. As a critical component of tumor immunosurveillance, impaired cytotoxicity of NK cells may promote the growth and metastasis of tumors 23-25. Second, opioids could directly interact with opioid receptors expressed by cancer cells, and stimulate tumor growth and metastasis 26. Overexpression of mu-opioid receptor (MOR) has been observed in human colorectal cancer samples 27. Activation of MOR by morphine promotes proliferation, invasion, and migration of human colorectal cancer cells, which is possibly due to transactivation of epidermal growth factor receptor (EGFR) and downstream signaling pathways 13.

In contrast, the association between decreased intraoperative opioid dose and worse recurrence-free survival (RFS) was observed in clinical studies of esophageal squamous cell carcinoma 5, and triple-negative breast cancer 28. Different levels of MOR expression and polymorphisms of the *OPRM1* gene may allow various cancer cells to respond differently to the stimulation of opioids 5. While in the study of triple-negative breast cancer, data of bulk RNA-seq were available and showed almost no expression of the *OPRM1* gene 28. This mechanism is not likely to be favorable in the case of CRLM, as increased expression of the *OPRM1* gene was observed in colorectal cancer tissue samples 27. A low level of perioperative equianalgesic fentanyl dose might be associated with an increased risk of patients receiving inadequate pain control. Increased surgical stress response and activation of the sympathetic nervous system, possibly the consequence of insufficient pain control, is considered to be immunosuppressive or promote invasiveness of tumor cells 29. Evaluation of the effectiveness of perioperative pain control, i.e. intraoperative hemodynamics and postoperative pain scoring, is lacking in this study, which limited further discussion.

In this study, we observed no statistically significant association between intraoperative equianalgesic fentanyl consumption and postoperative complications, PFS, or OS. These results were in line with a previous clinical study of intraoperative fentanyl consumption on prognosis of stage I-III colorectal cancer 4. While in another study of metastatic or recurrent colorectal cancer treated with therapeutic chemotherapy, the use of opioids was associated with worse outcomes 11. It is possible that long-term use of opioids for the management of cancer pain could involve higher cumulative dose of opioids compared to intraoperative opioids, and may lead to prolonged immunosuppression and worse prognosis of patients with colorectal cancer.

Several limitations are present in this study. First, results from analyses of the unmatched full cohort may be confounded by differences in baseline characteristics. The higher comorbidity burden and increased CEA of the low dose group could lead to worse outcomes of this group despite adjusted in multivariate analysis. While we tried to minimize confounding effect through PSM, the statistical power to tell the differences of outcomes between groups was reduced in the matched analysis. Second, data including NK cells activity, MOR expression, *OPRM1* polymorphism, and evaluation of perioperative pain control are lacking in the study and limited further discussion of possible mechanisms contributing to the results. As the inhibitory effect of opioids on NK cells last for several days, the opioid consumption in a short time period after surgery may also have prognostic importance 3, 6. Third, multiple anesthesiologists contributed to anesthesia and of patients in this study, and opioid doses prescribed by different anesthesiologists might be slightly biased. Finally, this is a retrospective cohort study with all patients treated at the same institution, and the results of this study require external validation.

## Conclusion

Intraoperative opioids consumption was not correlated with outcomes of CRLM patients treated with simultaneous resection.

## Figures and Tables

**Table 1:** Patient demographics, clinicopathological characteristics, and treatment details stratified by median intraoperative equianalgesic fentanyl dose

Group		Before PSM		*P*	After PSM		*P*
		Low dose (n=172)	High dose (n=171)		Low dose (n=55)	High dose (n=55)	
**Demographics & clinicopathological characteristics**							
Age		60.00 [52.75, 67.00]	58.00 [52.00, 64.00]	0.078	57.00 [50.00, 64.00]	58.00 [52.00, 64.00]	0.858
Gender	Male	53 (30.8)	65 (38.0)	0.161	15 (27.3)	17 (30.9)	0.675
	Female	119 (69.2)	106 (62.0)		40 (72.7)	38 (69.1)	
BMI		24.10 [22.57, 26.49]	23.31 [21.34, 25.66]	0.004	23.66 [22.13, 25.11]	23.71 [21.73, 25.63]	0.914
comorbidity	Yes	89 (51.7)	63 (36.8)	0.005	17 (30.9)	17 (30.9)	1
	No	83 (48.3)	108 (63.2)		38 (69.1)	38 (69.1)	
ASA	I	4 (2.3)	4 (2.3)	0.013	3 (5.5)	2 (3.6)	0.842
	II	138 (80.2)	155 (90.6)		49 (89.1)	49 (89.1)	
	III	30 (17.4)	12 (7.0)		3 (5.5)	4 (7.3)	
Primary site	Rectum	76 (44.2)	78 (45.6)	0.955	26 (47.3)	22 (40.0)	0.592
	Left colon	62 (36.0)	61 (35.7)		16 (29.1)	21 (38.2)	
	Right colon	34 (19.8)	32 (18.7)		13 (23.6)	12 (21.8)	
Bilobular distribution of liver metastasis	Yes	74 (43.0)	64 (37.4)	0.291	21 (38.2)	17 (30.9)	0.423
	No	98 (57.0)	107 (62.6)		34 (61.8)	38 (69.1)	
Number of liver metastasis		2.00 [1.00, 4.00]	2.00 [1.00, 4.00]	0.631	2.00 [1.00, 4.00]	2.00 [1.00, 4.00]	0.632
Maximum diameter of liver metastasis (cm)		2.50 [1.50, 3.80]	2.50 [1.50, 4.00]	0.492	2.50 [1.55, 3.65]	2.70 [1.50, 4.00]	0.467
Poor differentiation	Yes	59 (34.3)	60 (35.1)	0.879	19 (34.5)	16 (29.1)	0.539
	No	113 (65.7)	111 (64.9)		36 (65.5)	39 (70.9)	
Primary tumor T stage	T1-T2	14 (8.1)	16 (9.4)	0.69	5 (9.1)	6 (10.9)	0.751
	T3-T4	158 (91.9)	155 (90.6)		50 (90.9)	49 (89.1)	
Primary lymph node metastasis	Yes	128 (74.4)	125 (73.1)	0.781	42 (76.4)	38 (69.1)	0.392
	No	44 (25.6)	46 (26.9)		13 (23.6)	17 (30.9)	
CEA (ng/μL)		10.14 [5.32, 30.95]	6.75 [3.07, 23.95]	0.005	8.31 [4.40, 20.64]	8.39 [2.96, 32.56]	0.886
Extrahepatic metastasis	Yes	12 (7.0)	19 (11.1)	0.182	5 (9.1)	5 (9.1)	1
	No	160 (93.0)	152 (88.9)		50 (90.9)	50 (90.9)	
**Chemotherapy**							
Neoadjuvant chemotherapy	Yes	93 (54.1)	100 (58.5)	0.41	28 (50.9)	33 (60.0)	0.337
	No	79 (45.9)	71 (41.5)		27 (49.1)	22 (40.0)	
Adjuvant chemotherapy	Yes	116 (67.4)	104 (60.8)	0.201	39 (70.9)	33 (60.0)	0.229
	No	56 (32.6)	67 (39.2)		16 (29.1)	22 (40.0)	
**Surgical details**							
R0 resection	Yes	128 (74.4)	126 (73.7)	0.877	36 (65.5)	39 (70.9)	0.539
	No	44 (25.6)	45 (26.3)		19 (34.5)	16 (29.1)	
Intraoperative RFA	Yes	19 (11.0)	11 (6.4)	0.13	4 (7.3)	5 (9.1)	0.728
	No	153 (89.0)	160 (93.6)		51 (92.7)	50 (90.9)	
Surgical approach	Totally laparoscopic	45 (26.2)	40 (23.4)	0.586	16 (29.1)	13 (23.6)	0.627
	Mixed	87 (50.6)	96 (56.1)		23 (41.8)	28 (50.9)	
	Totally open	40 (23.3)	35 (20.5)		16 (29.1)	14 (25.5)	
Major hepatic resection	Yes	80 (46.5)	84 (49.1)	0.628	24 (43.6)	28 (50.9)	0.445
	No	92 (53.5)	87 (50.9)		31 (56.4)	27 (49.1)	
Intraoperative Pringle maneuver	Yes	124 (72.1)	131 (76.6)	0.338	39 (70.9)	42 (76.4)	0.516
	No	48 (27.9)	40 (23.4)		16 (29.1)	13 (23.6)	
Intraoperative blood loss (mL)		200.00 [100.00, 400.00]	200.00 [200.00, 400.00]	0.279	200.00 [100.00, 500.00]	200.00 [200.00, 300.00]	0.47
Intraoperative blood transfusion	Yes	39 (22.7)	43 (25.1)	0.592	16 (29.1)	15 (27.3)	0.832
	No	133 (77.3)	128 (74.9)		39 (70.9)	40 (72.7)	
Operation time (min)		324.50 [260.00, 410.25]	348.00 [269.00, 420.00]	0.451	310.00 [264.50, 380.50]	350.00 [266.50, 400.00]	0.453

**Table 2:** Short-term outcomes of patients stratified by median intraoperative equianalgesic fentanyl dose

Group		Before PSM		*P*	After PSM		*P*
		Low dose (n=172)	High dose (n=171)		Low dose (n=55)	High dose (n=55)	
**Postoperative Hospital stay**		10.00 [8.00, 13.25]	10.00 [8.00, 13.50]	0.841	11.00 [8.00, 15.00]	9.00 [8.00, 13.00]	0.199
Postoperative complication	No complication	83 (48.3)	93 (54.4)	0.099	24 (43.6)	29 (52.7)	0.077
	Minor complication	41 (23.8)	47 (27.5)		13 (23.6)	18 (32.7)	
	Major complication	48 (27.9)	31 (18.1)		18 (32.7)	8 (14.5)	
Postoperative complication category	No complication	83 (48.3)	93 (54.4)	0.615	24 (43.6)	29 (52.7)	0.272
	Surgery-related	31 (18.0)	31 (18.1)		14 (25.5)	13 (23.6)	
	General complication	30 (17.4)	23 (13.5)		4 (7.3)	7 (12.7)	
	Surgery-related and general complication	28 (16.3)	24 (14.0)		13 (23.6)	6 (10.9)	

**Table 3:** Univariate and Multivariate analysis of factors associated with progression-free survival and overall survival before PSM

		PFS				OS			
		Univariate HR	*P*	Multivariate HR	*P*	Univariate HR	*P*	Multivariate HR	*P*
Intraoperative equianalgesic fentanyl	Low dose	Referent		Referent		Referent		Referent	
	High dose	0.925 (0.720-1.19)	0.541	0.852 (0.655-1.11)	0.235	1.06 (0.732-1.53)	0.765	1 (0.68-1.49)	0.981
**Demographics & clinicopathological characteristics**									
Age		1.01 (0.991-1.02)	0.495			1.03 (1.01-1.05)	0.016	1.04 (1.02-1.06)	0.002
Gender	Male	Referent		Referent		Referent			
	Female	1.38 (1.05-1.8)	0.020	1.1 (0.825-1.46)	0.522	1.18 (0.797-1.74)	0.413		
BMI		1.02 (0.974-1.06)	0.478			1.02 (0.958-1.09)	0.507		
Comorbidity	No	Referent				Referent			
	Yes	1.09 (0.851-1.41)	0.486			1.07 (0.755-1.55)	0.725		
ASA	I	Referent				Referent			
	II	1.012 (0.476-2.15)	0.975			1.22 (0.387-3.86)	0.733		
	III	1.002 (0.44-2.28)	0.997			1.02 (0.288-3.62)	0.975		
Primary site	Rectum	Referent		Referent		Referent			
	Left colon	0.927 (0.703-1.22)	0.588	1.002 (0.753-1.33)	0.990	0.999 (0.664-1.5)	0.995		
	Right colon	0.733 (0.513-1.05)	0.089	0.711 (0.425-1.19)	0.193	0.943 (0.567-1.57)	0.822		
Bilobular distribution of liver metastasis	No	Referent		Referent		Referent		Referent	
	Yes	1.77 (1.38-2.28)	<0.001	0.91 (0.642-1.29)	0.596	1.64 (1.14-2.37)	0.009	0.666 (0.399-1.11)	0.119
Number of liver metastasis		1.15 (1.1-1.2)	<0.001	1.06 (0.989-1.13)	0.100	1.18 (1.12-1.25)	<0.001	1.13 (1.02-1.24)	0.015
Maximum diameter of liver metastasis (cm)		1.11 (1.04-1.18)	0.002	1.05 (0.971-1.13)	0.229	1.15 (1.05-1.26)	0.004	1.06 (0.957-1.18)	0.257
Poor differentiation	No	Referent		Referent		Referent		Referent	
	Yes	1.28 (0.986-1.67)	0.064	1.14 (0.87-1.5)	0.342	1.41 (0.959-2.07)	0.082	1.18 (0.787-1.77)	0.425
Primary tumor T stage	T1-T2	Referent		Referent		Referent		Referent	
	T3-T4	1.65 (1.01-2.7)	0.048	1.38 (0.832-2.3)	0.211	4.2 (1.33-13.2)	0.015	3.44 (1.06-11.1)	0.040
Primary lymph node metastasis	No	Referent		Referent		Referent		Referent	
	Yes	2.14 (1.56-2.94)	<0.001	1.84 (1.32-2.56)	<0.001	3.4 (1.91-6.07)	<0.001	3.04 (1.65-5.58)	<0.001
CEA (ng/μL)		1.001 (1-1.001)	0.070	1 (0.999-1.001)	0.989	1.001 (1-1.001)	0.298		
Extrahepatic metastasis	No	Referent		Referent		Referent			
	Yes	2.06 (1.38-3.070	<0.001	1.97 (1.3-2.99)	0.002	1.36 (0.744-2.47)	0.321		
**Chemotherapy**									
Neoadjuvant chemotherapy	No	Referent				Referent		Referent	
	Yes	1.02 (0.795-1.32)	0.855			1.48 (1.01-2.17)	0.046	1.19 (0.767-1.83)	0.444
Adjuvant chemotherapy	No	Referent				Referent		Referent	
	Yes	1.04 (0.797-1.35)	0.794			0.616 (0.425-0.893)	0.011	0.484 (0.324-0.725)	<0.001
**Surgical details**									
R0 resection	No	Referent		Referent		Referent		Referent	
	Yes	0.553 (0.421-0.727)	<0.001	0.686 (0.509-0.924)	0.013	0.556 (0.38-0.814)	0.003	0.84 (0.552-1.28)	0.416
Intraoperative RFA	No	Referent		Referent		Referent		Referent	
	Yes	1.71 (1.14-2.57)	0.010	1.25 (0.772-2.01)	0.369	2.16 (1.32-3.53)	0.003	1.08 (0.584-2.01)	0.802
Surgical approach	Totally laparoscopic	Referent		Referent		Referent		Referent	
	Mixed	1.52 (1.10-2.09)	0.011	0.981 (0.685-1.41)	0.918	1.58 (0.947-2.64)	0.080	1.03 (0.565-1.86)	0.934
	Totally open	1.24 (0.844-1.82)	0.273	1.04 (0.634-1.72)	0.867	1.62 (0.911-2.89)	0.101	1.04 (0.543-1.98)	0.913
Major hepatic resection	No	Referent		Referent		Referent		Referent	
	Yes	1.93 (1.5-2.49)	<0.001	1.49 (1.04-2.12)	0.029	2.21 (1.51-3.22)	<0.001	1.84 (1.06-3.19)	0.032
Intraoperative Pringle maneuver	No	Referent				Referent		Referent	
	Yes	1.18 (0.883-1.57)	0.265			1.51 (0.974-2.32)	0.066	0.646 (0.364-1.15)	0.137
Intraoperative blood loss (mL)		1.001 (1-1.001)	<0.001	1 (1-1.001)	0.772	1.001 (1-1.002)	0.007	1 (0.999-1.001)	0.893
Intraoperative blood transfusion	No	Referent				Referent		Referent	
	Yes	1.19 (0.891-1.58)	0.243			1.63 (1.09-2.44)	0.017	1.19 (0.679-2.1)	0.539
Operation time (min)		1.002 (1.001-1.003)	<0.001	1.001 (0.999-1.002)	0.330	1.003 (1.002-1.005)	<0.001	1.002 (1-1.004)	0.056

**Table 4:** Univariate and Multivariate analysis of factors associated with progression-free survival and overall survival after PSM

		PFS				OS			
		Univariate HR	*P*	Multivariate HR	*P*	Univariate HR	*P*	Multivariate HR	*P*
Intraoperative equianalgesic fentanyl	Low dose	Referent		Referent		Referent		Referent	
	High dose	0.996 (0.639-1.55)	0.984	1.05 (0.632-1.73)	0.861	1.31 (0.706-2.43)	0.392	1.74 (0.892-3.38)	0.105
**Demographics & clinicopathological characteristics**									
Age		1.003 (0.982-1.03)	0.764			1.04 (1.01-1.08)	0.016	1.06 (1.02-1.11)	0.003
Gender	Male	Referent		Referent		Referent			
	Female	1.68 (1.02-2.79)	0.047	1.23 (0.719-2.12)	0.446	1.17 (0.587-2.34)	0.653		
BMI		1.03 (0.948-1.12)	0.479			1.06 (0.939-1.19)	0.366		
Comorbidity	No	Referent				Referent			
	Yes	1.25 (0.779-1.99)	0.359			1.58 (0.822-3.04)	0.170		
ASA	I	Referent				Referent			
	II	1.26 (0.46-3.46)	0.652			3.15 (0.431-23)	0.258		
	III	0.609 (0.136-2.73)	0.517			6.73 (0.692-65.4)	0.101		
Primary site	Rectum	Referent				Referent		Referent	
	Left colon	0.718 (0.434-1.19)	0.197			0.808 (0.404-1.62)	0.546	0.67 (0.322-1.39)	0.284
	Right colon	0.668 (0.37-1.21)	0.181			0.482 (0.204-1.14)	0.098	0.429 (0.171-1.07)	0.070
Bilobular distribution of liver metastasis	No	Referent		Referent		Referent			
	Yes	1.59 (1.02-2.49)	0.043	1.06 (0.549-2.05)	0.860	1.24 (0.663-2.33)	0.497		
Number of liver metastasis		1.12 (1.02-1.22)	0.014	1.03 (0.918-1.15)	0.638	1.09 (0.97-1.22)	0.153		
Maximum diameter of liver metastasis (cm)		1.09 (0.973-1.22)	0.138			1.14 (0.973-1.34)	0.105		
Poor differentiation	No	Referent				Referent			
	Yes	1.23 (0.768-1.98)	0.386			1.13 (0.57-2.23)	0.730		
Primary tumor T stage	T1-T2	Referent				Referent			
	T3-T4	1.36 (0.627-2.97)	0.434			1.39 (0.427-4.5)	0.588		
Primary lymph node metastasis	No	Referent		Referent		Referent		Referent	
	Yes	2.33 (1.33-4.11)	0.004	2.42 (1.34-4.36)	0.004	2.45 (1.03-5.83)	0.043	2.83 (1.14-7.02)	0.025
CEA (ng/μL)		1.002 (0.999-1.005)	0.146			1 (0.997-1.01)	0.613		
Extrahepatic metastasis	No	Referent		Referent		Referent			
	Yes	2.3 (1.13-4.68)	0.023	2.23 (1.07-4.67)	0.033	1.21 (0.428-3.42)	0.721		
**Chemotherapy**									
Neoadjuvant chemotherapy	No	Referent		Referent		Referent			
	Yes	0.676 (0.434-1.05)	0.082	0.588 (0.364-0.951)	0.031	1.26 (0.674-2.37)	0.467		
Adjuvant chemotherapy	No	Referent				Referent			
	Yes	1.06 (0.664-1.7)	0.804			0.657 (0.352-1.23)	0.187		
**Surgical details**									
R0 resection	No	Referent				Referent			
	Yes	0.815 (0.509-1.31)	0.394			0.859 (0.454-1.62)	0.639		
Intraoperative RFA	No	Referent				Referent			
	Yes	1.33 (0.609-2.89)	0.477			1.48 (0.583-3.77)	0.415		
Surgical approach	Totally laparoscopic	Referent				Referent			
	Mixed	1.01 (0.591-1.74)	0.959			1.48 (0.653-3.33)	0.349		
	Totally open	0.792 (0.426-1.47)	0.461			1.25 (0.508-3.05)	0.632		
Major hepatic resection	No	Referent		Referent		Referent		Referent	
	Yes	1.59 (1.02-2.48)	0.042	1.28 (0.704-2.34)	0.415	2.24 (1.2-4.2)	0.012	1.49 (0.715-3.1)	0.287
Intraoperative Pringle maneuver	No	Referent				Referent		Referent	
	Yes	1.27 (0.762-2.1)	0.363			2.1 (0.968-4.56)	0.061	1.09 (0.424-2.78)	0.865
Intraoperative blood loss (mL)		1.001 (1-1.002)	0.057	1.001 (1-1.002)	0.105	1.001 (1-1.002)	0.028	1.001 (1-1.003)	0.035
Intraoperative blood transfusion	No	Referent				Referent			
	Yes	1.14 (0.704-1.84)	0.598			1.63 (0.861-3.09)	0.133		
Operation time (min)		1.002 (1.001-1.004)	0.007	1.001 (0.999-1.003)	0.486	1.003 (1.001-1.005)	0.004	1.002 (0.999-1.005)	0.118

## Abbreviations

NK: natural killer
CRLM: colorectal liver metastasis
PFS: progression-free survival
OS: overall survival
PSM: propensity score matching
IQR: interquartile ranges
BMI: body mass index
ASA: American Society of Anesthesiologists
RFA: radiofrequency ablation
NSCLC: non-small-cell lung cancer
MOR: mu-opioid receptor
EGFR: epidermal growth factor receptor
RFS: recurrence-free survival
